# Flora volatile profiles of plants visited by *Vespa velutina*: a preliminary assessment in the interaction of plant-insect

**DOI:** 10.1007/s10265-025-01645-5

**Published:** 2025-05-15

**Authors:** M. Shantal Rodríguez-Flores, Ana Diéguez-Antón, M. Carmen Seijo-Coello, Olga Escuredo

**Affiliations:** Department of Plant Biology and Soil Sciences, Universidade de Vigo, Ourense, 32004 Spain

**Keywords:** Natural phytochemicals, Plant volatiles, Terpenoids, *Vespa velutina*

## Abstract

Plants function within complex ecological communities, relying on chemical signals to mediate interactions with other organisms. The foraging behaviour of insects, such as the invasive hornet *Vespa velutina nigrithorax*, introduced into northwestern Spain over a decade ago, may be influenced by floral volatiles. This hornet detects plant secondary metabolites, including semiochemicals, which aid in locating nectar, carbohydrates, prey, mating sites, and other resources. Understanding the volatile organic compounds (VOCs) emitted by plants visited by *V. velutina* may help to develop targeted control strategies. The aim of this study was to identify and analyse the volatile compounds emitted by 18 plant species frequented by *V. velutina nigrithorax* in the province of Ourense, northwest Spain. Solid-phase microextraction (SPME) coupled with gas chromatography-mass spectrometry (GC-MS) was used in this study. A total of 110 VOCs were identified, of which 21 compounds were abundant in the samples, with terpenes being the most abundant. Furthermore, a PLS-DA analysis selected 33 volatile compounds with variable importance scores (VIPs) greater than 1, in particular methylanthranilate with a value of 1.81. Eleven of these compounds were found to be abundant in the analysed samples, including (*Z*)-β-ocimene; 1-octen-3-ol; 3-hexen-1-ol, acetate, (*Z*)-; 3-octanone; eugenol; linalool; methyl salicylate; *o*-cymene; α-farnesene; α-terpineol and β-farnesene. The selection of these compounds provides valuable insights into plant-insect interactions, highlighting their diverse roles as plant volatiles in mediating insect behaviour and underlining their potential as targets for environmentally friendly pest management strategies.

## Introduction

Communication between plants and animals is essential for their proper development. Animals use a variety of signals, including acoustic, electrical, magnetic, visual and chemical cues. Among the chemical signals, semiochemicals play a key role in mediating insect behaviour (Abd El-Ghany 2020). These compounds can affect different species, such as allelochemicals, or affect the same species, such as pheromones (Abd El-Ghany 2020; El-Ghany 2019). Plant biochemical processes release a wide array of volatile chemicals into the environment. Volatile organic compounds (VOCs) are critical for plant adaptation to the environment and serve as infochemicals in multitrophic interactions. They contribute to various ecological functions, including indirect plant defence against insects, pollinator attraction, plant-plant communication, plant-pathogen interactions, and scavenging of reactive oxygen species, as well as thermotolerance and other stress adaptations (Yuan et al. 2009). Over 1,000 volatile chemicals have been identified in plants, a number expected to grow with advances in detection and analytical techniques (Pichersky et al. 2006). Most plant volatiles are lineage-specific and function in specialized ecological interactions, classifying them as specialized or secondary metabolites (Pichersky et al. 2006). Advances in analytical techniques, along with molecular and biochemical methods, have made plant volatiles among the most well-studied secondary metabolites. Particular attention has been given to volatile terpenoids (Cheng et al. 2007; Zwenger Chhandak Basu et al. 2008). The finding that plant volatiles transmit specific information about herbivorous insects has unveiled a sophisticated and complex chemically mediated interaction between plants and insects. Plants can release chemicals that serve as important feeding signals to attract the natural enemies of herbivores, thereby enhancing their defences against herbivorous insects. For example, females of the parasitoid wasp *Cotesia marginiventris* have learned to recognize plant volatiles with hosts or host by-products, enabling them to locate prey (Turlings et al. 1991). This synthesis and release of chemical signals is an active physiological process, often triggered by herbivores oral secretions (De Moraes et al. 2001). Despite progress, the study and application of plant VOCs remains in early stages. Further research is needed to unravel the synergistic and/or antagonistic effects of biotic and abiotic factors on plant VOCs release from plants (Liu et al. 2023).

Invasive and exotic species can threaten agricultural economies, global food security, and human livelihoods. Introduced species become invasive when they establish and proliferate across ecosystems, disrupting biodiversity, ecosystem services, and human welfare (Bellard et al. 2016; Simberloff et al. 2013). The spread of the invasive species *Vespa velutina nigrithorax* poses a critical challenge to the conservation of biodiversity and the ecosystem integrity. This hornet is an effective predator of honey bees and other pollinating insects, raising concerns about its potential to disrupt the delicate plant-pollinator interactions that are an essential component of the natural systems that maintain ecosystem functions and services. By disrupting pollination processes, *V. velutina* directly threatens native species that depend on pollination for reproduction, seed dispersal and fruit production (Traveset and Richardson 2006). There are therefore serious implications for pollination ecosystem services, such as crop pollination services for food production and human livelihoods. This species has not only important environmental impacts but also economic and social implications.

Due to its severe impact on various sectors, control of *V. velutina* is critical to mitigate its ecological, economic and social impacts. To date, management efforts have primarily relied on baiting methods using non-selective chemical attractants. These control methods are challenging due to their multiple negative impacts on the environment and local entomofauna (Rodríguez-Flores et al. 2019). The bait-trapping method typically employs a commercial or homemade attractant that is non-specific and captures a wide range of insects, particularly Diptera, but also wasps, bees, bumblebees, moths, ants, lacewings, beetles, arachnids, and other species that are important for the ecosystem regulation (Rodríguez-Flores et al. 2019). Furthermore, several researchers have emphasized that these traps are not effective in reducing the number of nests in the treated areas (Beggs et al. 2011; Goldarazena et al. 2015; Laurino et al. 2020; Monceau and Thiéry 2017). The insects caught in these traps are mostly different from those consumed by the hornet. This is the case for the European hornet, *Vespa crabro*, which is caught in the same traps used for *V. velutina*. Therefore, the use of the traps studied so far exerts additional pressure compared to the natural pressure exerted by *V. velutina* (Rodríguez-Flores et al. 2019; Rojas-Nossa et al. 2018). The development of ecological alternatives has become a priority and an important objective in our society. Some studies have focused on advancing environmentally friendly pest management strategies, including the potential use of semiochemicals, such as pheromones, as a means of controlling this pest (Couto et al. 2014; Gévar et al. 2017; Rodríguez-Flores et al. 2021a, b; Wen et al. 2017). Semiochemicals offer the potential to provide sustainable control alternatives to the use of synthetic insecticides (Rodríguez-Flores et al. 2021b). Methods include the use of insect attractants or stimulants, arrestants, repellents and deterrents. Plant semiochemicals are known to elicit wide range of behavioural responses in insects. Consequently, these compounds are plant volatiles (PVs) with allelochemical functions (Zhang et al. 2016) and may serve as an alternative control method.

*V. velutina* is a eusocial hornet species that communicates using semiochemicals. These compounds along with other chemical components distributed across the hornet’s body, perform multiple functions related to colony organisation (Richard and Hunt 2013; Rodríguez-Flores et al. 2021a). Most biological processes in this species are regulated by semiochemicals, making these compounds potential attractants for bait-trapping. This is the case for feeding or foraging in *V. velutina*. The yellow-legged hornet builds its nest based on the resources available in the environment. It has been observed that visual and olfactory cues can influence different aspects of social recognition, such as prey location by *V. velutina* (Couto et al. 2017; Wang et al. 2014). The flora surrounding the nest constitutes the main food source for this species. The larvae of this species require large amounts of protein for their development, while adults feed on a carbohydrates-rich diet i obtained from sugary liquids such as plant nectar. Flower nectar is one of the main sources of sugar, and hornets are often observed visiting flowers to feed on nectar (Ueno 2015). This provides them with the energy required for long-distance flights and prey searching. The attractiveness of these resources is linked to the recognition of olfactory cues that can be detected over long distances. Most of the studies on the diet of this hornet have focused on its protein intake derived from insect species, including other pollinators, with a particular emphasis on honey bees (Diéguez-Antón et al. 2022; Requier et al. 2019; Rojas-Nossa and Calviño-Cancela 2020; Rome et al. 2021). However, information on the plant species with which *V. velutina* interacts remains scarce (Rojas-Nossa et al. 2023).

In the northwest of the Iberian Peninsula, *V. velutina* has become well established, interacting with the biodiversity present in the region. Among the species it exploits for resources are several flora species, some of which are associated with carbohydrate acquisition at the beginning of its biological cycle and/or serve as mating sites in autumn. In this context, the present study aimed to provide the volatile composition of 18 plants where individuals of *V. velutina* were frequently observed foraging for insects or collecting nectar. The geographic area sampled was located in the inland region of Galicia (northwestern Spain). This study represents the first attempt in Spain to identify specific volatile compounds from commonly occurring plants that may attract *V. velutina*.

## Materials and methods

### Plant selection and sampling

**Fig. 1:**
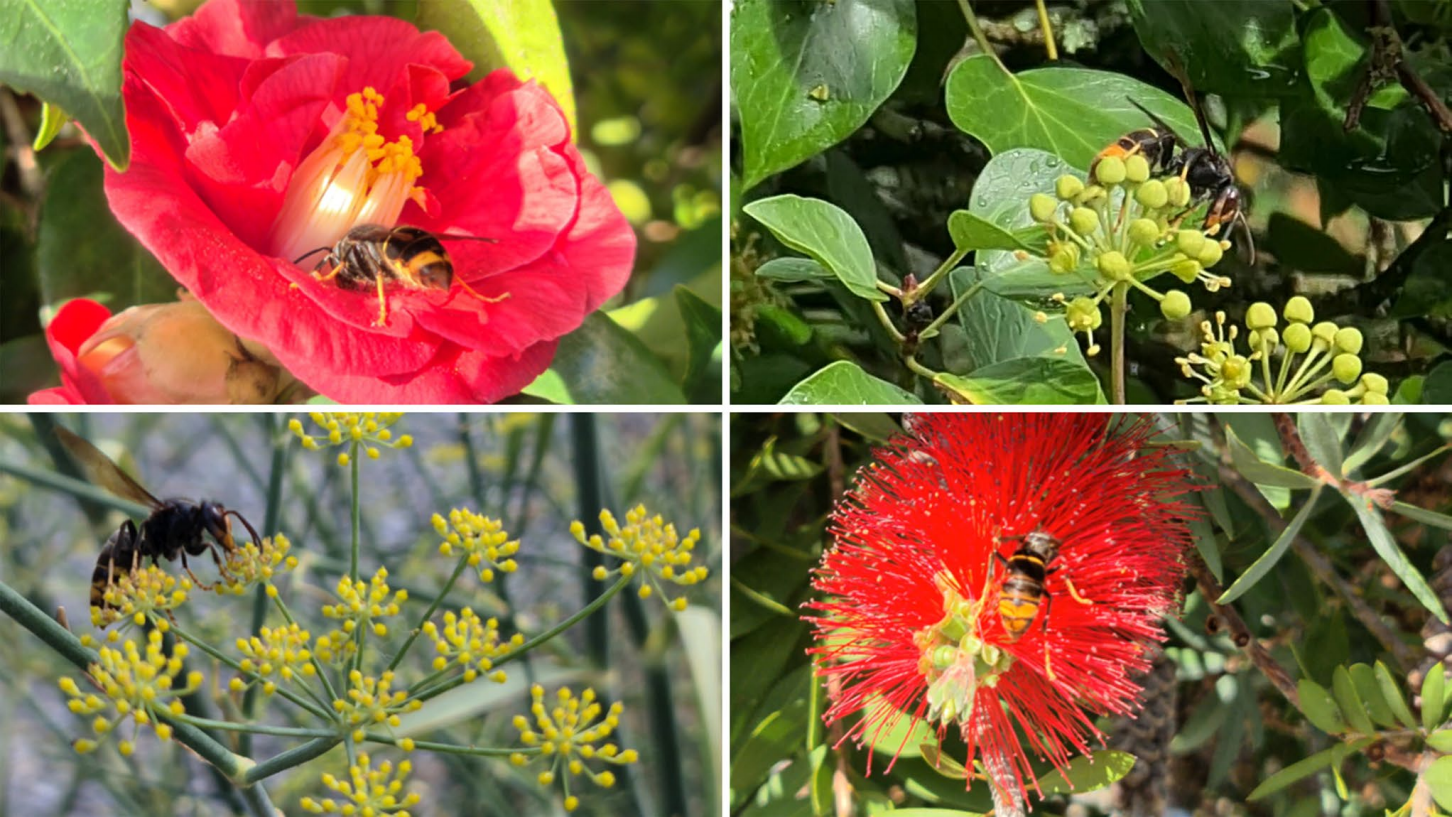
*V. velutina* feeding on different plant species (*Camellia japonica* and *Hedera helix*, at the top, *Foeniculum vulgare*, and *Callistemon citrinus* in the bottom)

A total of 106 records of *V. velutina* were obtained through field observations conducted in 12 localities in the province of Ourense (northwestern Spain). Plants were selected based on two main criteria: (1) consistent and repeated visits from *V. velutina* over time, and (2) a high numbers of hornets per plant, with some species even being used as trap sites due to their strong attractiveness to the hornet. Some of these interactions have already been reported in previous studies, supporting the selection criteria (Rojas-Nossa and Calviño-Cancela 2020; Ueno 2015). Examples of these plant-hornet interactions are shown in Fig. 1. Following this protocol, 18 plant species were identified as being frequently visited by the hornet in search of food. These plants were selected for volatile profile analysis (Table 1).

**Table 1:** Classification of the plant species most visited by *V. velutina*

Family	Species	Common names
Apiaceae	*Foeniculum vulgare* Mill.	Fennel
	*Oenanthe crocata* L.	Hemlock water-dropwort
Araliaceae	*Hedera helix* L.	Ivy
Boraginaceae	*Echium vulgare* L.	Viper’s bugloss
Brassicaceae	*Brassica oleracea* L.	Wild cabbage.
	*Brassica rapa* L.	Rape
Fabaceae	*Robinia hispida* L.	Bristly locust
	*Robinia pseudoacacia* L.	Black locust
Lamiaceae	*Lamium maculatum* L.	Spotted deadnettle
	*Lavandula stoechas* L.	Spanish lavender
	*Mentha suaveolens* Ehrh.	Apple mint
Lythraceae	*Punica granatum* L.	Pomegranate
Malvaceae	*Tilia americana* L.	American basswood
Meliaceae	*Melia azedarach* L.	Chinaberry tree
Myrtaceae	*Callistemon citrinus* (Curtis) Dum.Cours.	Crimson bottlebrush
Oleaceae	*Ligustrum vulgare* L.	Common privet
Scrophulariaceae	*Buddleja davidii* Franch.	Butterfly-bush
Theaceae	*Camellia japonica* L.	Japanese camellia

Among the plants visited, the most common families were Fabaceae and Lamiaceae. Other families, such as Brassicaceae, Campanulaceae, Ericaceae and Malvaceae, were also frequently recorded. Within these groups, the most frequently visited species were *C. japonica*,* H. helix*,* M. suaveolens*, and *F. vulgare*. The sampling period spanned from October 25, 2020, to October 29, 2021. All plant samples were collected in the morning (around 10 a.m.). The material was then transported to the laboratory for volatile extraction, with no more than 30 min elapsing between collection and extraction.

### Extraction of volatile compounds of plants

Flowers that attracted *V. velutina* were collected in the field. A minimum of 2 g of whole, undisturbed, freshly picked flowers was immediately immersed in a 50 mL vial containing a 30% sodium chloride solution at a 1:2 (w/v) ratio. The solution was continuously stirred and heated to 50 °C, and VOC extraction was initiated within 20 min of collection to preserve the natural volatile profile. Volatile compounds were extracted by using solid-phase microextraction (SPME). SPME overcomes the shortcomings of traditional sample pretreatment techniques. The advantage of this technology lies in the integration of sampling, extraction, concentration and injection, which greatly reduces sample volume and cost, thereby accelerating analysis and detection. In this study, a fiber coated with a 65 μm film of polydimethylsiloxane/divinylbenzene (PDMS/DVB) (Supelco SPME fiber 57326U, Darmstadt, Germany) was used. All samples were analysed in duplicate.

### Gas chromatography mass spectrometry (GC-MS) analysis

The SPME fiber was exposed to the vapor phase of the vial for 60 min with agitation to capture volatiles. The fiber was then withdrawn and transferred to the gas chromatograph (GC) injector in splitless mode for 5 min, where the volatiles were desorbed. A Thermo Finnigan Trace GC Ultra gas chromatograph (San Jose, CA), equipped with a Thermo Finnigan Trace DSQ selective mass detector and a ZB-5MSi capillary column (30 m x 0.25 mm, thickness 0.25 μm; Zebron Phenomenex, Torrance, USA) was used to separate the volatiles. The internal temperature was programmed from 40 °C to 170 °C (3 °C/min), then from 170 °C to 290 °C (25 °C/min), holding 290 °C for 15 min. Helium was used as the carrier gas at a constant flow rate of 1 mL/min. The mass spectra were acquired at an ionization energy of 70 eV, in full-scan mode over a mass range from m/z 35 to 500. The transfer line and ion source temperature were set at 250 °C and 230 °C, respectively.

### Data acquisition and volatile compound identification

Data acquisition was performed using Xcalibur.ink software. Compound identification was carried out using the NIST 2011 mass spectral library. To confirm compound identification, Linear Retention Indices (LRIs) were calculated for each detected compound. The LRI values were determined using a mixture of n-alkanes (C_7_–C_40_) (Supelco, Bellefonte, PA, USA) dissolved in hexane, applying the following formula:

$$\:{I}_{x}={100}_{n}+100\frac{({t}_{x}-{t}_{n})}{({t}_{n+1}-{t}_{n})}$$

Where:

I_x_ is the Linear retention index of the compound to be identified.

𝑛 is the carbon number of the nearest alkane with a shorter retention time.

𝑡_𝑥_ is the retention time of the unknown compound.

𝑡_𝑛_ is the retention time of the alkane with 𝑛 carbon atoms.

𝑡_𝑛+1_ is the retention time of the alkane with _𝑛+1_ carbon atoms.

Relative area values (expressed as the percentage of total volatiles) were obtained directly from the Total Ion Chromatogram (TIC).

### Statistical analysis

Data were processed using Studio (version 2024.12.0 + 467; Posit Software, PBC), with R (version 3.6.0). A PLS-DA (Partial Least Squares Discriminant Analysis) was performed using the kernel-based PLS fitting method (kernelpls) to identify the VOCs that contributed most to distinguishing among plant species. The model generated 15 components, with the first two explaining 94.7% of the variance in the species variable. Variable Importance in Projection (VIP) scores were calculated for each predictor variable using the following formula:

$$\:VIPj=\sqrt{p\times\:\frac{\sum\:_{h}\left({w}_{jh}^{2}{.SS}_{h}\right)}{\sum\:SS}}$$

where:

W_jh_ are the loading weights of variable j in component h.

SS_h_ is the sum of squares explained by component h.

## Results

### Main volatile organic compounds identified

A total of 110 volatile organic compounds were extracted from 18 living plants using the solid-phase microextraction (SPME) technique. The volatile compounds were classified into 20 chemical categories based on the PubChem Compound Database from the National Center for Biotechnology Information (NCBI), including acetate esters, alcohols, aldehydes, alkenes, benzyl compounds, benzene derivatives, benzoate esters, esters, ethers, fatty acid esters, fatty acid ethyl esters, fatty acid methyl esters, furans, isothiocyanates, ketones, monoterpenes, nitrogen-containing compounds, oxygenated analogues sesquiterpenes, sesquiterpenes, and sulfur compounds. Most of these compounds exhibit functional similarities to those of aromatic hydrocarbons (Table 2).

**Table 2:** Classification of the identified compounds into the various categories, with the LRI calculated and the LRI theoretical

Group	Compound	CAS RN	LRI	LRIt
Acetate esters	3-Hexen-1-ol, acetate, (*Z*)-	3681-71-8	1009	1009
	4-Hexen-1-ol, acetate, (*Z*)-	42125-17-7	1009	1001
	Nerol acetate	141-12-8	1366	1364
	Bornyl acetate	76-49-3	1291	1291
Alcohols	1-Nonen-3-ol	21964-44-3	1081	1088
	3-Nonanol	624-51-1	1097	1099
	3-Octanol	589-98-0	997	991
	2-Hexen-1-ol;	2305-21-7	863	854
	1-Octen-3-ol	3391-86-4	980	976
	Benzyl alcohol	100-51-6	1035	1031
	3-Hexen-1-ol, (*Z*)-	928-96-1	852	858
	3-Hexen-1-ol, (*E*)-	928-97-2	855	855
	1-Hexanol	111-27-3	865	865
	1-Hexanol, 2-ethyl-	104-76-7	1028	1029
	Phenylethyl Alcohol	60-12-8	1116	1114
	2-Octen-1-ol, (*Z*)-	26001-58-1	1070	1066
	2-Heptanol	543-49-7	899	900
Aldehydes	Nonanal	124-19-6	1105	1105
	Phenylacetaldehyde	122-78-1	1046	1049
	Benzaldehyde	100-52-7	963	966
	2-Hexenal, (*E*)-	6728-26-3	848	850
Alkenes	Cyclohexene, 1-methyl-5-(1-methylethenyl)-, (R)-	1461-27-4	1032	1028
Bencyl compounds	2-(4-Methylphenyl)propan-2-ol	1197-01-9	1189	1175
	Eugenol	97-53-0	1363	1359
	Anethole	4180-23-8	1300	1301
Benzene Derivatives	1,2-Dimethoxybenzene	91-16-7	1148	1149
	Benzene, 1,4-dimethoxy-	150-78-7	1166	1165
	1,3,5-Trimethoxybenzene	621-23-8	1414	1418
Benzoate esters	Benzoic acid, ethyl ester	93-89-0	1174	1172
	Benzoic acid, methyl ester	93-58-3	1097	1094
	Methyl anthranilate	134-20-3	1346	1346
	Methyl salicylate	119-36-8	1199	1197
	Ethyl salicylate	118-61-6	1275	1270
Esters	Valeric acid, 3-methylbut-2-enyl ester	80039-51-6	1147	n.f.
Ethers	Estragole	140-67-0	1205	1201
Fatty acid esters	2-methylbutyl 3-methylbutanoate	2445-77-4	1109	1110
	*cis*-3-Hexenyl butyrate	16491-36-4	1189	1187
	*cis*-3-Hexenyl iso-butyrate	41519-23-7	1145	1145
Fatty acid ethyl esters	Ethyl nonanoate	123-29-5	1299	1300
Fatty acid methyl esters	Octanoic acid, methyl ester	111-11-5	1126	1128
Furans	2-Pentylfuran	3777-69-3	991	996
Isothiocyanates	3-Butenyl isothiocyanate	3386-97-8	984	982
	4-Methylpentyl isothiocyanate	17608-07-0	1169	1166
	Allyl Isothiocyanate	57-06-7	883	887
	Cyclopentyl isothiocyanate	33522-03-1	1085	n.f.
Ketones	Dibenzyl ketone	102-04-5	1211	n.f.
	Dihydrooxophorone	20547-99-3	1170	1170
	2,6,6-Trimethyl-2-cyclohexene-1,4-dione	1125-21-9	1147	1152
	2-Heptanone	110-43-0	888	893
	3-Octanone	106-68-3	987	985
	5-Hepten-2-one, 6-methyl-	110-93-0	988	991
	3-Cyclopenten-1-one, 2-hydroxy-3-(3-methyl-2-butenyl)-	69745-70-6	1376	1389
	Geranyl acetone	689-67-8	1457	1456
	Piperitenone	491-09-8	1349	1343
	Piperitone oxide	5286-38-4	1260	1259
	2-Nonanone	821-55-6	1093	1096
Monoterpenes	Sabinene	3387-41-5	975	976
	α-Terpineol	98-55-5	1195	1195
	(-)-Camphor	464-48-2	1151	1146
	(+)-Isopiperitenone	16750-82-6	1278	1271
	(*Z*)-β-Ocimene	3338-55-4	1050	1050
	2-Thujene	28634-89-1	976	978
	Camphene	79-92-5	950	952
	Cyclohexane, 2-ethenyl-1,1-dimethyl-3-methylene-	95452-08-7	1118	1121
	Eucalyptol	470-82-6	1035	1031
	Isopinocarveol	6712-79-4	1144	1148
	Fenchone	126-21-6	1096	1092
	Limonene oxide, *cis*-	13837-75-7	1138	1138
	Linalool oxide	1365-19-1	1074	1071
	*trans*-Sabinenehydrate	17699-16-0	1071	1060
	α-Myrcene	1686-30-2	993	986
	γ-Terpinene	99-85-4	1061	1057
	α-Fenchene	471-84-1	951	950
	o-Cymene	527-84-4	1027	1024
	β-*trans*-Ocimene	3779-61-1	1046	1046
	Linalool	78-70-6	1102	1103
	α-Pinene	80-56-8	936	937
	β-Pinene	127-91-3	979	978
	β-Terpinene	99-84-3	1063	1056
	L-α-Terpineol	10482-56-1	1193	1192
	Linalyl acetate	115-95-7	1259	1248
	(-)-Terpinen-4-ol	20126-76-5	1182	1182
	β-Myrcene	123-35-3	991	991
	Borneol	507-70-0	1171	1173
	d-Carvone	2244-16-8	1249	1257
	Fenchyl acetate	138-51-11-1	1239	1232
	Limonene	138-86-3	1032	1027
	Terpinolene	586-62-9	1091	1089
	D-sylvestrene	1461-27-4	1031	1032
Nitrogen-containing compounds	Formamide, N-phenyl-	103-70-8	1220	1220
Oxygenated analogues sesquiterpenes	+/-*trans*-Nerolidol	40716-66-3	1579	1567
Sesquiterpenes	Humulene	6753-98-6	1466	1463
	α-Farnesene	502-61-4	1515	1511
	α-Gurjunene	489-40-7	1419	1412
	β-Elemene	515-13-9	1400	1394
	β-Farnesene	18794-84-8	1461	1459
	γ-Elemene	29873-99-2	1440	1440
	γ-Muurolene	30021-74-0	1485	1479
	δ-Elemene	20307-84-0	1343	1342
	α-Cubebene	17699-14-8	1366	1366
	(-)-Zingiberene	495-60-3	1520	1513
	Elemene isomer	08/08/3242	1345	1344
	(-)-γ-Elemene	30824-67-0	1510	1511
	Alloaromadendrene	25246-27-9	1451	1453
	Caryophyllene	87-44-5	1431	1425
	*cis*-β-Farnesene	28973-97-9	1459	1458
	Copaene	3856-25-5	1383	1372
	Germacrene D	23986-74-5	1491	1486
Sulfur compounds	Dimethyl trisulfide	3658-80-8	971	970
	Disulfide, methyl (methylthio)methyl	42474-44-2	1129	1134

n.f.: not found; LRI, linear retention index determined on a ZB-5MSi column relative to a series of n-alkanes (C_7_–C_40_); LRI: Linear retention index theoretical obtained through the NIST Chemistry Web Book, SRD 69; CAS RN: CAS Registry Number

**Fig. 2:**
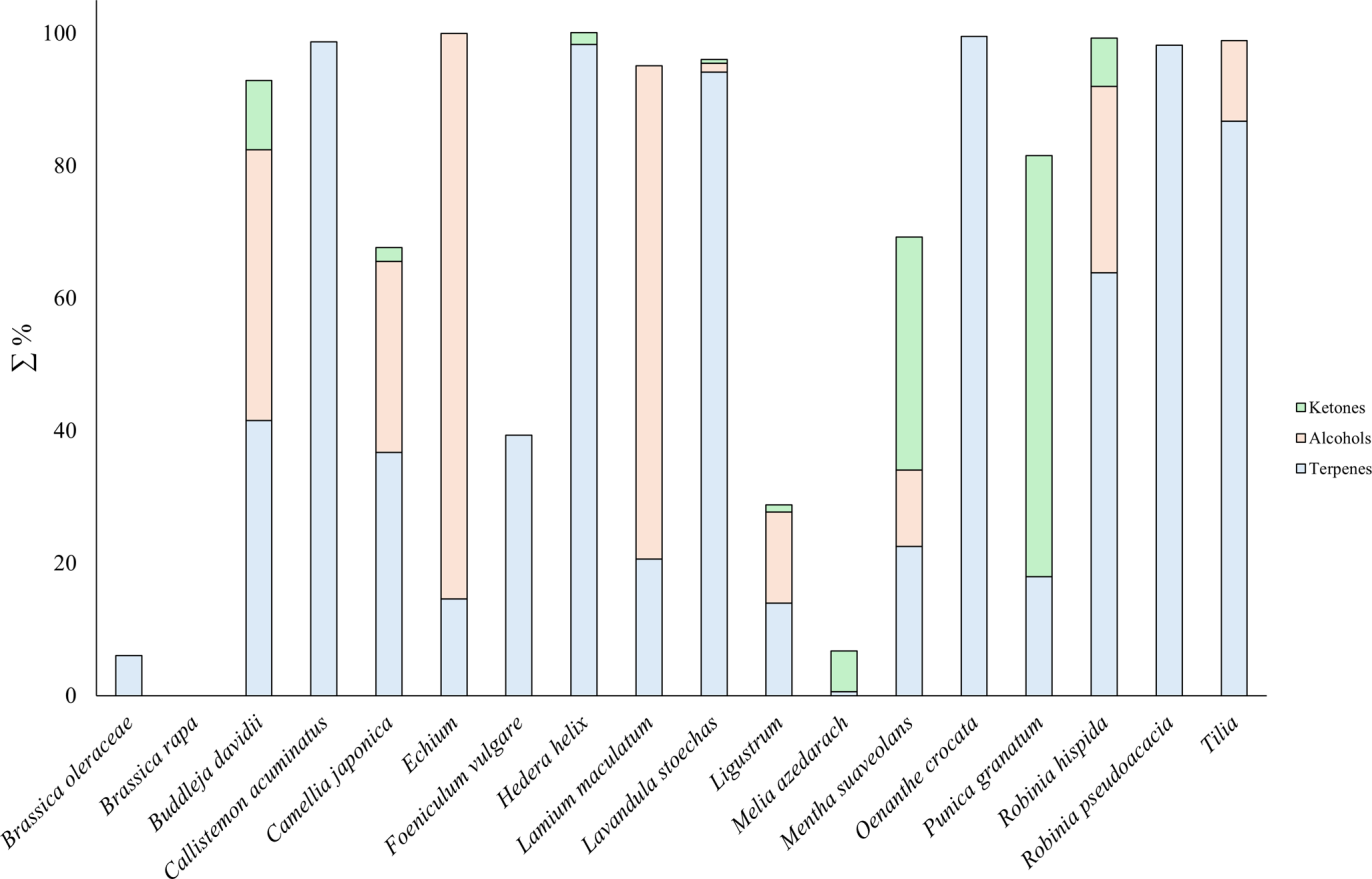
Total content of the most abundant chemical groups in each plant species studied

The most prevalent compounds were of the terpene type, including monoterpenes and sesquiterpenes. In total, 33 monoterpenes and 17 sesquiterpenes were identified. Additionally, alcohol (13 compounds), and ketones (11 compounds) were identified as frequent compounds (Fig. 2). The remaining categories exhibited a lower number of compounds, with fewer than five compounds identified in each category.

### Main composition of each plant species

Volatile compounds are presented for 18 plant species belonging to 13 botanical families. The main volatile compounds, along with their respective relative concentrations, are presented in Table 3. The different plant species were classified according to their taxonomic families.

**Table 3:** Main composition and their mean relative concentration (%) of each plant species, classified by family

Family	Species	Compounds [%]
Apiaceae	*Foeniculum vulgare*	Anethole (44.8%); Fenchone (31.4%); Estragole (13.0%); Cyclohexene, 1-methyl-5-(1-methylethenyl)-, (R)-(12.2%); β-Pinene (5.5%); β-Myrcene (1.4%); γ-Terpinene (1.0%); Fenchyl acetate (0.1%)
	*Oenanthe crocata*	(*Z*)-β-Ocimene (71.4%); β-*trans*-Ocimene (13.5%); β-Terpinene (7,1%); Sabinene (4.7%); *o*-Cymene (0.8%); β-Myrcene (0.7%); Linalool (0.6%); Germacrene D (0.5%); 2-methylbutyl 3-methylbutanoate (0.4%); β-Farnesene (0.2%)
Araliaceae	*Hedera helix*	γ-Muurolene (21.3%); (-)-Zingiberene (19.4%); Germacrene D (14.4%); γ-Elemene (12.2%); *cis*-β-Farnesene (8.0%); β-Myrcene (6.6%); δ-Elemene (5.5%); α-Pinene (4.2%); β-Pinene (3.1%); β-*trans*-Ocimene (2.0%); D-sylvestrene (1.7%); Dihydrooxophorone (0.8%); 2,6,6-Trimethyl-2-cyclohexene-1,4-dione (0.8%); 5-Hepten-2-one, 6-methyl- (0.2%)
Boraginaceae	*Echium vulgare*	3-Hexen-1-ol, (*E*)- (56.1%); 2-Hexen-1-ol- (27.3%); Eucalyptol (9.6%); 1-Octen-3-ol (2.0%); α-Terpineol (1.8%); 2-Thujene (1.7%); α-Myrcene (1.5%)
Brassicaceae	*Brassica oleraceae*	Allyl isothiocyanate (78.7%); Disulfide, methyl (methylthio)methyl (7.2%); Eucalyptol (6.0%); Dimethyl trisulfide (1.1%)
	*Brassica rapa*	3-Butenyl isothiocyanate (87.9%); Cyclopentyl isothiocyanate (9.9%); Phenylacetaldehyde (0.9%); 3-Hexen-1-ol, acetate, (*Z*)- (0.7%); 4-Methylpentyl isothiocyanate (0,4%); Nonanal (0.1%); *cis*-3-Hexenyl iso-butyrate (0.1%); Octanoic acid, methyl ester (0.1%)
Fabaceae	*Robinia hispida*	Cyclohexane, 2-ethenyl-1,1-dimethyl-3-methylene- (30.4%); 1-Octen-3-ol (26.4%); (*Z*)-β-Ocimene (14.4%); β-Farnesene (11.6%); 3-Octanone (7.3%); Linalool (6.1%); α-Farnesene (1.4%); 3-Octanol (1.2%); Methyl salicylate (0.7%); Benzyl alcohol (0.5%)
	*Robinia pseudoacacia*	(*Z*)-β-Ocimene (65.3%); Linalool (30.6%); β-Myrcene (2.3%); Methyl anthranilate (1.8%)
Lamiaceae	*Lamium maculatum*	1-Octen-3-ol (66.1%); 2-Thujene (7.6%); Linalyl acetate (6.5%); Methyl salicylate (4.0%); 3-Hexen-1-ol, (*E*)- (3.1%); 2-Octen-1-ol, (*Z*)- (2.8%); 3-Octanol (2.5%); Germacrene D (2.2%); Linalool (1.6%); β-Myrcene (1.6%); Benzaldehyde (0.9%); Humulene (0.7%); α-Farnesene (0.7%)
	*Lavandula stoechas*	Fenchone (29.2%); Germacrene D (21.2%); (-)-Camphor (20.0%); β-Pinene (11.2%); Linalool (3.4%); α-Cubebene (2.6%); Camphene (2.5%); 3-Octanol (1.3%); Bornyl acetate (1.3%); Copaene (1.3); α-Gurjunene (0.9%); Eucalyptol (0.9%); β-Myrcene (0.7%); 3-Octanone (0.6%); Borneol (0.3%); Nerol acetate (0.1%)
	*Mentha suaveolens*	3-Cyclopenten-1-one, 2-hydroxy-3-(3-methyl-2-butenyl)- (34.5%); *trans*-Sabinenehydrate (12.3%); 1-Octen-3-ol (6.4%); 3-Octanol (4.9%); (-)-Terpinen-4-ol (2.2%); β-Myrcene(1.8%); 2-Thujene (1.0%); Linalool (0.7%); Borneol (0.6%); Caryophyllene (0.6%); β-elemene (0.6%); (+)-Isopiperitenone (0.5%); Humulene (0.5%); α-Terpineol (0.5%); 3-Octanone (0.5%); Methyl salicylate (0.3%); *o*-Cymene (0.3%); β-Farnesene (0.3%); Eucalyptol (0.2%); Piperitone oxide (0.2%); Eugenol (0.1%); α-Fenchene (0.1%); 2-(4-Methylphenyl)propan-2-ol (0.1%); (*Z*)-β-Ocimene (0.1%); Valeric acid, 3-methylbut-2-enyl ester (0.1%); Isopinocarveol (0.1%); 3-Nonanol (0.1); Phenylethyl alcohol (0.1%); 1-Nonen-3-ol (0.1%); Piperitenone (0.1%); d-Carvone (0.05%); Phenylacetaldehyde (0.04%); Limonene oxide, *cis*- (0.02%)
Lythraceae	*Punica granatum*	2-Nonanone (63.6%); Linalool (18.0%); Cyclohexene, 1-methyl-5-(1-methylethenyl)-, (R)- (15.2%); Eugenol (2.5%)
Malvaceae	*Tilia americana*	Terpinolene (47.2%); *o*-Cymene (30.1%); Phenylethyl alcohol (12.2%); β-Myrcene (5.2%); (-)-Terpinen-4-ol (2.8%); 2-(4-Methylphenyl)propan-2-ol (1.1%); Eucalyptol (0.9%); d-Carvone (0.5%)
Meliaceae	*Melia azedarach*	+/-*trans*-Nerolidol (72.9%); Benzene, 1,4-dimethoxy- (8.7%); Formamide, N-phenyl- (6.4%); 5-Hepten-2-one, 6-methyl- (6.2%); Nonanal (4.3%); Phenylacetaldehyde (0.8%); Linalool (0.6%)
Myrtaceae	*Callistemon citrinus*	Eucalyptol (38.9%); α-Pinene (19.9%); Limonene (8.8%); Caryophyllene (8.2%); (-)-γ-Elemene (7.2%); Elemene isomer (4.8%); β-Myrcene (2.6%); Alloaromadendrene (2.6%); (*Z*)-β-Ocimene (2.0%); α-Terpineol (2.0%); Linalool (1.6%); 3-Hexen-1-ol, acetate, (*Z*) (0.9%); α-Cubebene (0.3%)
Oleaceae	*Ligustrum vulgare*	*cis*-3-Hexenyl butyrate (23.9%); Benzoic acid, ethyl ester (16.5%); Linalool (14.0%); Phenylethyl Alcohol (13.8%); 1,3,5-Trimethoxybenzene (13.2%); 4-Hexen-1-ol, acetate, (*Z*)- (4.5%); Phenylacetaldehyde (4.2%); Ethyl salicylate (3.3%); 1,2-Dimethoxybenzene (2.5%); Benzoic acid, methyl ester (1.9%); Dibenzyl ketone (1.1%); Nonanal (1.0%); Ethyl nonanoate (0.2%)
Scrophulariaceae	*Buddleja davidii*	α-Farnesene (41.6%); 1-Octen-3-ol (34.6%); 2,6,6-Trimethyl-2-cyclohexene-1,4-dione (8.5%); Methyl salicylate (4.9%); Benzyl alcohol (2.4%); Eugenol (2.2%); Phenylethyl alcohol (2.1%); 3-Octanol (1.8%); 3-Octanone (1.5%); Geranyl acetone (0.4%)
Theaceae	*Camellia japonica*	Linalool (19.6%); Phenylacetaldehyde (16.0%); L-α-Terpineol (14.7%); 2-Heptanol (8.1%); 2-Hexen-1-ol; (5.5%); 2-Hexenal, (*E*)- (5.3%); Phenylethyl alcohol (5.1%); *cis*-3-Hexen-1-ol (3 7%); 1-Hexanol (2.4%); 2-Heptanone (2.2%); 2-Pentylfuran (2.1%); 1-Hexanol, 2-ethyl (1.9%); 1-Octen-3-ol (1.7%); Linalool oxide (1.5%); Eucalyptol (1.2%); Nonanal (1.0%); Bornyl acetate (1 0%); 3-Hexen-1-ol, acetate, (*Z*)- (0.7%); Benzyl alcohol (0.6%)

On examination of the concentration data, six compounds were found to be present at concentrations greater than 60% in the samples. One of the compounds with the highest concentration was 3-butenyl isothiocyanate, which was present at an average of 87% in *B. rapa*. It is noteworthy that another species of the Brassicaceae family, such as *B. oleracea*, also presented another isothiocyanate with high percentages, such as allyl isothiocyanate, with an average relative concentration of 78.7%. Another compound with a concentration greater than 60% was the sesquiterpene +/-*trans*-nerolidol, with an average relative concentration of 72.9% in *M. azedarach*. This was followed by the monoterpene (*Z*)-β-ocimene, with an average value of 71.4% in *Oenanthe crocata* and 65.3% in *R. pseudoacacia*. In addition, 1-octen-3-ol, with a mean relative concentration of 66.1% in *L. maculatum* and the ketone compound 2-nonanone with a mean concentration of 63.6% in *Punica granatum*, presented high relative concentrations. The remaining compounds exhibited concentrations below the aforementioned threshold in the plant samples.

Considering the species with the highest number of compounds, *M. suaveolens* samples exhibit a particularly noteworthy profile, with an average of 34 volatile compounds. As a result, the family with the highest number of compounds was Lamiaceae. The volatile profile revealed that the ketone 3-cyclopenten-1-one, 2-hydroxy-3-(3-methyl-2-butenyl) was the most abundant compound (34.5%), followed by the monoterpene *trans*-sabinenehydrate (12.3%) and 1-octen-3-ol (6.4%). The remaining plant samples exhibited a lower number of compounds, with *C. japonica* being the sample with the highest number of volatile compounds after *M. suaveolens*. A total of 20 volatile compounds were identified as constituting the volatile profile, with the monoterpene linalool being the compound with the highest relative concentration (13.5%). The volatile profile of *L. stoechas* was found to comprise 16 compounds. The most abundant VOCs were the terpene compounds fenchone (29.2%), and germacrene D (21.2%), followed by (-)-camphor (20.0%). *H. helix* showed the presence of 14 volatile organic compounds (VOCs), with γ-muurolene (21.3%) and (-)-zingiberene (19.4%) sesquiterpenes representing the most abundant compounds. The volatile profiles of the samples of *C. citrinus*,* L. maculatum*, and *L. vulgare* exhibited a total of 13 different volatile compounds. For *C. citrinus*, the monoterpene eucalyptol was the most abundant volatile compound with a concentration of 38.9%, followed by the monoterpenes α-pinene (19.9%) and limonene (8.8%), and the sesquiterpene caryophyllene (8.1%). In *L. maculatum*, as already mentioned, the alcohol 1-octen-3-ol was the most volatile compound, followed by the monoterpenes 2-thujene with an average concentration of 7.6%, and linalyl acetate (6.4%). In *L. vulgare*, the carboxylic acid *cis*-3-hexenyl butyrate presented the highest concentration with an average value of 23.9%. This was followed by benzoic acid, ethyl ester (16.5%); linalool (14%), phenylethyl alcohol (13.8%) and the aromatic ether 1,3,5-trimethoxybenzene (13.2%).

A total of 10 volatile compounds were identified in *B. davidii*, *O. crocata*, and *Robinia hispida*. In *B. davidii*, the sesquiterpene α-farnesene (41.6%) and 1-octen-3-ol (34.6%) were the most abundant VOCs in the volatile profile. (*Z*)-β-Ocimene was identified as the volatile compound with the highest concentration in *O. crocata*, representing 71.4% of the total VOCs present. The main compound identified in *R. hispida* was the monoterpene cyclohexane, 2-ethenyl-1,1-dimethyl-3-methylene- (30.4%), followed by 1-octen-3-ol (26.4%) and (*Z*)-β-ocimene (14.4%).

Eight compounds were identified in the samples of *B. rapa*, *F. vulgare*, and *T. americana*. The isothiocyanates 3-butenyl isothiocyanate (87.9%) and cyclopentyl isothiocyanate (9.9%) were the most representative in the samples of *B. rapa*, while anethole (44.8%) and fenchone (31.4%) were the most abundant in the samples of *F. vulgare*, and *o*-cymene (30.0%), phenylethyl alcohol (12.2%) and the monoterpene terpinolene (47.2%) in *T. americana*. Seven volatile compounds were identified in the *E. vulgare* and *M. azedarach* samples. Although no compound stood out with very high concentrations in *E. vulgare*, a hexanol 3-hexen-1-ol, (*E*)- with mean values of 56.1% and an alkenyl alcohol 2-hexen-1-ol- with mean value of 27.3% were presented as main compounds. While in *M. azedarach* as mentioned above, the main compound was +/-*trans*-nerolidol with a high value of 72.9%. For *P. granatum* sample, five different compounds were identified. Among the identified volatile compounds, 2-nonanone was found to be the predominant component with a mean value of 63.6%.

The profiles of *Brassica oleracea* and *R. pseudoacacia* were less comprehensive, with only four compounds identified in each case. In addition to isothiocyanate detected in *B. oleracea*, the monoterpene eucalyptol (6.0%) and the sulfur compounds dimethyl trisulfide (1.1%) and disulfide, methyl (methylthio)methyl (7.2%) were also identified. In the case of *R. pseudoacacia*, in addition to (*Z*)-β-ocimene (65.3%), the monoterpene linalool exhibited a mean concentration of 30.6%, the monoterpene β-myrcene 2.3%, and the aminobenzoate methyl anthranilate 1.8%.

### Most frequent and important compounds in the sampled plants

**Fig. 3:**
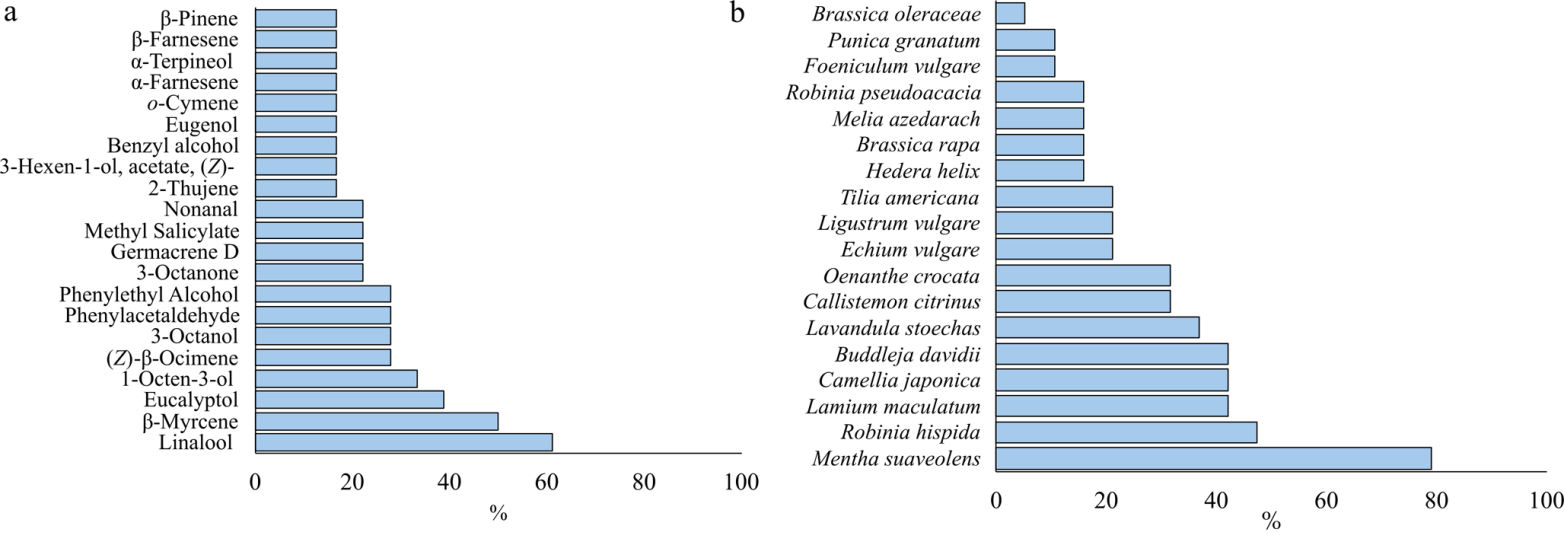
Bar chart showing (**a**) % presence of the compounds in at least three of the species under study; (**b**) % of most abundant compounds in each species

A total of 21 compounds were identified as the most frequent among the 110 compounds that were identified (Fig. 3a, b). The selection of frequent VOCs was determined as those detected in a minimum of three of the species under investigation. Among these, 10 compounds were identified as terpenes, comprising eight monoterpenes ((*Z*)-β-ocimene-; 2-thujene; eucalyptol; linalool; α-terpineol; β-myrcene and β-pinene) and three sesquiterpenes (β-farnesene; α-farnesene; and germacrene D). Other commonly occurring compounds included the acetate ester 3-hexen-1-ol, acetate (*Z*); the four alcohols 1-octen-3-ol; 3-octanol; benzyl alcohol and phenylethyl alcohol; the two aldehydes nonanal and phenylacetaldehyde; the benzoate ester methyl salicylate; the two benzyl compounds eugenol and *o*-cymene; and the ketone 3-octanone.

Among compounds present in a larger number of samples (Fig. 3a), linalool and β-myrcene were identified in over 50% (*N* = 9) of the plant species. Eucalyptol and 1-octen-3-ol were present in more than 30% (*N* = 6) of the plants studied. Eight compounds ((*Z*)-β-ocimene, 3-octanol, phenylacetaldehyde, phenylethyl alcohol, 3-octanone, germacrene D, methyl salicylate and nonanal) were present in more than four species. The compounds 2-thujene; 3-hexen-1-ol, acetate, (*Z*)-; benzyl alcohol; eugenol; *o*-cymene; α-farnesene; α-terpineol; β-farnesene and β-pinene were identified in three plant species. The remaining identified compounds were detected in a single plant species.

Across the studied species, *M. suaveolens*, exhibited the highest proportion of representative compounds (78.9%) (Fig. 3b), following by *R. hispida* (47.4%). *L. maculatum*, *C. japonica* and *B. davidii*, accounted for 42.1% of these compounds, while *L. stoechas* (36.8%) and *C. citrinus* and *O. crocata* (31.6% each). A lower proportion of representative compounds (21.1%) were identified in *E. vulgare*, *L. vulgare*, and *T. americana*. Only 15.8% of the compounds were detected in four species (*H. helix*, *B. rapa*, *M. azedarach* and *R. pseudoacacia*). The lowest percentage of these compounds was observed in *F. vulgare* and *P. granatum* (10.5%), followed by *B. oleraceae*, which showed only one compound among the most representative ones (eucalyptol).

In addition, a Partial Least Squares Discriminant Analysis (PLS-DA) analysis was conducted to evaluate the role of volatile organic compounds (VOCs) in distinguishing among plant species. The analysis yielded Variable Importance in Projection (VIP) scores for each compound, with scores exceeding 1 indicating significant contributions to species differentiation. Table 4 shows the most important compounds contributing to species differentiation. As presented in Table 4 and 33 compounds had VIP scores greater than 1, with methyl anthranilate exhibiting the highest VIP score of 1.81. This suggests the importance of these VOCs in differentiating the plant species. Among these, eleven compounds were common to those frequently found in the samples in the previous section ((*Z*)-β-ocimene; 1-octen-3-ol; 3-hexen-1-ol, acetate, (*Z*)-; 3-octanone; eugenol; linalool; methyl salicylate; *o*-cymene; α-farnesene; α-terpineol and β-farnesene). Other compounds were also identified from the PLS-DA analysis, some of which were important compounds in the samples or unique to the species, and thus became key variables for species discrimination (allyl isothiocyanate, dimethyl trisulfide, methyl (methylthio)methyl disulfide, 2-hexen-1-ol, 3-hexen-1-ol, (*E*)-, α-myrcene, 2-methylbutyl 3-methylbutanoate, sabinene, β-terpinene, geranylacetone, and linalyl acetate). The alcohols 2-hexen-1-ol and 3-hexen-1-ol, (*E*)- were found to be present in significant quantities in *E. vulgare*. Furthermore, the monoterpene α-myrcene was detected exclusively in *E. vulgare*. Methyl anthranilate occurred exclusively in *R. pseudoacacia*. 2-Methylbutyl 3-methylbutanoate, sabinene and β-terpinene were identified as the exclusive compounds in *O. crocata*. Allyl isothiocyanate, the primary volatile organic compound (VOC), in conjunction with dimethyl trisulfide, and disulfide, methyl (methylthio)methyl, was exclusively present in *B. oleracea*. 2-Nonanone was identified as the most significant compound in *P. granatum*. Geranylacetone was exclusive to *B. davidii*. Cyclohexane, 2-ethenyl-1,1-dimethyl-3-methylene was important in *R. hispida* while linalyl acetate was detected exclusively in *L. maculatum.* Terpinolene was predominant and exclusive to *T. americana*.

**Table 4:** Variable importance projection (VIP) scores of VOCs above the threshold of 1.00 obtained from partial least squares discriminant analysis (PLS-DA) to discriminate between different plant species

Variable	VIP score
Methyl anthranilate	1.81
2-nonanone	1.63
Linalool	1.53
Cyclohexane, 2-ethenyl-1,1-dimethyl-3-methylene-	1.47
β-Farnesene	1.47
Allyl Isothiocyanate	1.45
Disulfide, methyl (methylthio)methyl	1.45
Dimethyl trisulfide	1.45
3-Octanone	1.41
(*Z*)-β-Ocimene	1.28
α-Myrcene	1.28
2-Hexen-1-ol	1.28
3-Hexen-1-ol, (*E*)-	1.27
Cyclohexene, 1-methyl-5-(1-methylethenyl)-, (R)-	1.20
Eugenol	1.19
2-methylbutyl 3-methylbutanoate	1.13
Sabinene	1.13
β-Terpinene	1.13
β-*trans*-Ocimene	1.11
o-Cymene	1.10
Terpinolene	1.10
d-Carvone	1.09
2-(4-Methylphenyl)propan-2-ol	1.08
Methyl Salicylate	1.06
2,6,6-Trimethyl-2-cyclohexene-1,4-dione	1.06
Geranylacetone	1.04
1-Octen-3-ol	1.04
α-Farnesene	1.03
3-Hexen-1-ol, acetate, (*Z*)-	1.01
Benzaldehyde	1.01
Linalyl acetate	1.01
2-Octen-1-ol, (*Z*)-	1.01
α-Terpineol	1.00

## Discussion

The mutualistic relationship between nectar-producing plants and the hornet *V. velutina* has received limited attention. Hornets are generally considered opportunistic flower visitors (Rojas-Nossa et al. 2023; Ueno 2015). However, this relationship may be more complex than previously assumed. Interactions between hornets and plants may extend beyond predation of other insects, as certain flora may also serve as carbohydrate sources, providing essential energy for vital physiological functions. For example, in spring, hornets have been observed consuming nectar from flowering flora such as *Camellia*, which is necessary for the establishment of a new colony (Monceau et al. 2014). The presence of partially digested pollen has been demonstrated in the digestive system of *V. velutina* larvae, corresponding to specific plant species (Diéguez-Antón et al. 2023). This suggests a closer association between this plant species and *V. velut*ina, beyond incidental pollen ingestion via predation of pollinators. Some plant species visited by *V. velutina* have previously been identified as potential food sources: *Callistemon* (Ueno 2015), *C. japonica*, *H. helix* (Monceau et al. 2014; Ueno 2015), and *M. suaveolens* (Rojas-Nossa and Calviño-Cancela 2020; Ueno 2015). Consequently, some of these species, such as the camellia, the crimson bottlebrush, and the ivy, have been employed as sites for the bait-trapping of queens. In the present study, 18 plant species were added to the list of flora visited by *V. velutina* (*C. citrinus*,* C. japonica*,* H. helix*,* M. suaveolens*,* B. oleraceae*,* B. rapa*,* B. davidii*,* E. vulgare*,* F. vulgare*,* L. maculatum*,* L. stoechas*,* L. vulgare*,* M. azedarach*,* O. crocata*,* P. granatum*,* R. hispida*,* R. pseudoacacia*, and *T. americana*). Many of these have been previously studied as valuable resources for pollinators, owing to their abundance of sugar-rich nectar and protein-rich pollen. Examples include *T. americana* (Jacquemart et al. 2018), C. *japonica* (Rho and Choe 2003), *E. vulgare* (Chwil and Weryszko-Chmielewska 2011), and other species recognised for their role in honey production (*Punica*, *Foeniculum*, *Brassica* (Homrani et al. 2020), *Echium*, *Lavandula* (Seijo et al. 2019). These floral resources likely act as reward following attraction by specific volatile organic compounds that mediate insect-plant interactions. However, the interaction of plant chemical signals on insects can result in the stimulation or inhibition of behavioural responses. Depending on the specific chemical signals involved, it can elicit an attractive or aversive response. Volatile compounds therefore play an essential role in attracting pollinators.

Phytochemical research on plant compounds has led to the isolation and identification of various active compounds, facilitating the exploration of their diverse future applications. These compounds hold significant potential for the monitoring and control of insect pests, either as standalone interventions or as components of integrated pest management strategies. Most are produced by plants as secondary metabolites, serving a wide range of functions (Zwenger Chhandak Basu et al. 2008). Among the most abundant groups of these metabolites are the terpenes (Wink 2010), which exhibit a broad spectrum of biological activities, including the attraction and repulsion of insects (Gershenzon and Dudareva 2007). Terpenes are typically volatile and form key constituents of essential oils. They play a crucial role in the attraction of specific pollinators and are also involves in insect pheromones systems and other chemical signals. Therefore, the application of terpenes, either synthesised in vitro or expressed in transgenic plants, as biopesticides has become a focal point in current and future research projects (Cheng et al. 2007; Zwenger Chhandak Basu et al. 2008).

In this study, eight monoterpenes (terpinolene, (*Z*)-β-ocimene, 2-thujene, eucalyptol, linalool, α-terpineol, β-myrcene, β-pinene) and 3 sesquiterpenes (germacrene D, α-farnesene, and β-farnesene) showed remarkable results due to their presence across multiple plants samples. The chemical diversity of terpenes in plants is influenced by their physiological and developmental states. Terpenes have been demonstrated to serve as key chemical mediators in both abiotic and biotic interactions. Some of the aforementioned terpenes have been studied for their attractant properties, suggesting their potential utility in pest management strategies. Thus, considering their attractant activity, terpinolene and *o*-cymene, identified as the primary compounds of *T. americana* in this study, are monoterpenes that are commonly found in insect-attractive volatile blends (Lanne et al. 1987; Saraiva et al. 2022). The acyclic monoterpene β-ocimene, detected in high relative concentrations in the samples of *O. crocata* and *R. pseudoacacia*, has been shown to act as a general attractant for a broad spectrum of pollinators (Farré-Armengol et al. 2017). The attractive properties of some of them have been studied in other organisms, such as fungi. The monoterpene 2-thujene, found in *E. vulgare*, *L. maculatum*, and *M. suaveolens*, has also been identified as a volatile compound of *Beauveria b*assiana, an entomopathogenic fungus that may attract insect pests such as *Myzus persicae* (Geedi et al. 2023), and *Coptotermes formosanus* (Hussain et al. 2010). This compound has also been highlighted as a potential biological control agent against *V. velutina*, given the fungus’s natural parasitism of the pest (Poidatz et al. 2018, 2019). Certain monoterpenes have demonstrated synergistic attractiveness when combined. Thus, the monoterpenes eucalyptol, α-pinene and fenchone, detected in *C. citrinus* and *F. vulgare* have been associated with the attraction of *Aegorhinus superciliosus*, underlining their potential application in integrated pest management programmes (Tampe et al. 2020). In addition, the eucalyptol has been found to attract other species such as the redbay ambrosia beetle (*Xyleborus glabratus*) (Kendra et al. 2016), and the female wasp of *Anagrus nilaparvatae* (Mao et al. 2018) in various blends. However, eucalyptol alone can also attract other insects such as the beetle *X. glabratus* (Kuhns et al. 2014). Other monoterpenes and aromatic alcohols, such as linalool, α-terpineol, and the benzyl alcohol, identified in *C. japonica* and other species, have been reported as components of yellowjacket wasp attractant system (Aldrich et al. 1985, 2004).

Linalool (3,7-dimethyl-1,6-octadien-3-ol), in particular, is a floral volatile integral to plant-pollinator interactions and has recently been shown to mediate the complex balance between pollinator attraction and plant defence, which is mediated by linalool and its derivatives in a diverse array of plants (Raguso 2016; Zhang et al. 2023). Its consistent detection across plants visited by *V. velutina* suggests its potential as a bait candidate for attracting this invasive hornet. β-myrcene, another monoterpene found in several samples (*F. vulgare*, *O. crocata*, *H. helix*, *R. pseudoacacia*, *L. maculatum*,* L. stoechas*,* M. suaveolens*,* T. americana*, and *C. citrinus*), is typically known for its repellent properties (Sun et al. 2020). However, it can also act as an attractant when combined with other compounds, such as phenylacetaldehyde. This combination has proven highly attractive to several pest species including *Argyrogramma verruca*, *Mocis disseverans*, *Heliothis virescens*, *Spodoptera eridania*, several noctuid moths, and *Diaphania hyalinata* (Meagher and Landolt 2008). Lastly, β-pinene, identified in several plants of this study (*F. vulgare*, *H. helix*, and *L. stoechas*) has been studied as a component of field traps for predaceous insects in cotton fields. In combination with α-pinene or alone, it can significantly attract the green lacewing *Chrysoplera sinica*, *Orius* spp. and *Chrysopa* spp (Yu et al. 2018). While beneficial for enhancing predator populations in agricultural settings, this non-specific attraction poses a limitation in bait-trapping strategies targeting *V. velutina*, as it may inadvertently lure non-target predatory insects, diminishing the bait’s effectiveness.

Sesquiterpenes are also constituents of essential oils and resins of plants. They have also been studied for their allelopathic properties, such as germacrene D. This is considered as a precursor of numerous sesquiterpene hydrocarbons (Bülow and König 2000). Germacrene D has been identified in various organisms and is an essential component in species such as fig and walnut (Buttery et al. 1986; Nawade et al. 2020; Tahara et al. 1975). It also serves as a known pheromone in cockroach (Tahara et al. 1975). In the present study, germacrene D was found in *O. crocata*, *H. helix*, *L. maculatum*, and *L. stoechas*. Its occurrence in certain plant species has been examined in relation to its influence on insect behaviour, for instance in fig trees and their interaction with the black fig fly *Silba adipata* (Nawade et al. 2020). Two other widely studied sesquiterpenes, α-farnesene and β-farnesene, were found in six plants species analysed in this study (*O. crocata*, *H. helix*,* R. hispida*,* L. maculatum*,* M. suaveolens*, and *B. davidii*). These compounds are also known for their allelopathic function. Both are produced by various plant species and are incorporated into the pheromonal communication of different insects. For instance, α-farnesene, is a component of the alarm pheromone in *V. velutina* (Rodríguez-Flores et al. 2021), while (*E*)-β-Farnesene is a well-known aphid alarm pheromone (Pickett and Griffiths 1980). These compounds act as an effective infochemicals used by insects to locate prey and hosts. (*E*)-β-Farnesene, for instance, is detected by the ladybird *Adalia bipunctata* to locate aphid prey (Francis et al. 2004), whereas α-farnesene can be discerned by the parasitoid wasp *Microplitis croceipes* to identify its host (Ngumbi et al. 2012). Given these properties, the potential role of α-farnesene and β-farnesene in either attracting or repelling *V. velutina* warrants further investigation, with a view to their possible application in trap-based control strategies.

The results of this study have led to the identification of additional volatile compounds in the plants under analysis, beyond those belonging to the terpene family. The most frequently occurring compounds include acetate esters (3-hexen-1-ol, acetate, (*Z*)-), alcohol compounds (1-octen-3-ol, 3-octanol, benzyl alcohol, phenylethyl alcohol), aldehydes (nonanal and phenylacetaldehyde), benzyl compounds (eugenol and *o*-cymene), benzoate esters (methyl salicylate), and ketones (3-octanone). The combination of these volatile compounds with other compounds, including terpenes, has been investigated as an integrated pest management strategies for various insect pests. The alkenyl alcohol 1-Octen-3-ol, identified in *E. vulgare*, *R. hispida*,* L. maculatum*,* M. suaveolens* and *B. davidii*, is produced by several plant species, as well as fungi (Kamiński et al. 1980; Pierce et al. 1991; Pyysalo 1976). Its attractant properties, both alone and in combination with other fungal-emitted compounds such as 3-octanol and 3-octanone, have been evaluated for their effectiveness in attracting various insect pests, including cucujids (Pierce et al. 1991). Furthermore, the attractiveness of 1-octen-3-ol has been assessed in other pest control applications, for instance, in the control of biting flies when combined with carbon dioxide (Logan and Birkett 2007), and in the capture of the invasive wasp species *Vespula vulgaris* when combined with 2-phenylethyl acetate, and methyl salicylate (Brown et al. 2015).

Among the aldehydes, nonanal is of particular interest due to its prior detections in living *V. velutina* specimens by HS-SPME/GC-MS (Couto et al. 2017; Rodríguez-Flores et al. 2021). In this study, nonanal has been identified in four plants: *B. rapa*, *M. azedarach*, *L. vulgare*, and *C. japonica*. As a saturated fatty aldehyde, nonanal plays a communicative role in wasp and hornet species and is recognized by *V. velutina* (Couto et al. 2017), suggesting its potential utility in bait-trapping strategies targeting this invasive species.

Eugenol, a carboxylic acid found in various plants, was detected in *M. suaveolens*, *P. granatum*, and *B. davidii*. The attractiveness of this compound has been demonstrated as a sex pheromone component identified in *Anagrus atomus*, which has been shown to successfully attract males of this species (Zanolli et al. 2021).

As a result of PLS-DA analysis, a total of 33 compounds were identified with VIP scores greater than 1, thereby highlighting them as potential compounds for further study. A number of these compounds have been previously discussed in the preceding paragraphs, including linalool; β-farnesene; 3-octanone; (*Z*)-β-ocimene; eugenol; *o*-cymene; methyl salicylate; 1-octen-3-ol; α-farnesene; 3-hexen-1-ol, acetate, (*Z*)- and α-terpineol. Among the most noteworthy compounds, methyl anthranilate and 2-nonanone showed the highest VIP values in this analysis. Methyl anthranilate was detected exclusively in *R. pseudoacacia*. This compound, a widespread floral volatile, is known for its potent kairomonal activity involvement in numerous ecological interactions. It has been shown to attract various insect species, including flower thrips and their parasitoid wasp *Ceranisus menes* (Imai et al. 2001). Additionally, braconid wasps have demonstrated responsiveness to this compound (James 2005).

2-Nonanone, a volatile ketone identified as the most abundant compound in *P. granatum*, has previously been documented as a component of alarm pheromones and venom volatiles in *V. velutina* (Cappa et al. 2019; Wen et al. 2017; Rodríguez-Flores et al. 2021a; Couto et al. 2017). In terms of kairomonal activity, 2-nonanone may serve as an interspecific chemical cue. Given its established role in *V. velutina* chemical ecology, it warrants further investigation as a potential attractant in integrated pest management strategies.

The PLS-DA also revealed a diverse set of additional volatile compounds, including allyl isothiocyanate, dimethyl trisulfide, methyl (methylthio)methyl disulfide, 2-hexen-1-ol, (*E*)-3-hexen-1-ol, α-myrcene, 2-methylbutyl 3-methylbutanoate, sabinene, β-terpinene, geranylacetone, and linalyl acetate. Allyl isothiocyanate, a glucosinolate hydrolysis product known for its defensive role in cruciferous plants, may act as a kairomone, aiding host location recognition by specialist herbivores (Fahey et al. 2001). Similarly, dimethyl trisulfide and methyl (methylthio)methyl disulfide, sulfur volatile compounds characteristic of Brassicaceae, have been implicated in plant–insect interactions (Jagodič et al. 2017; Kugimiya et al. 2010).

In *E. vulgare*, significant amounts of 2-hexen-1-ol, (*E*)-3-hexen-1-ol, and α-myrcene were detected. 2-Hexen-1-ol and 3-hexen-1-ol (*E*) are known mediators of plant defence and signalling, acting as chemical signals (kairomones) under certain conditions (Ruther et al. 2002; Yan et al. 2023). In plant-insect interactions, α-myrcene may influence insect behaviour. For example, α-myrcene, in combination with hexanal, s-limonene, phytol and carvacrol, has been shown to elicit stronger responses in males than in females *Phthorimaea operculella* (potato moth) (Das et al. 2007).

In *O. crocata*, 2-methylbutyl 3-methylbutanoate, sabinene, and β-terpinene were identified. 2-methylbutyl 3-methylbutanoate, a major volatile from the Brindley glands of *Triatoma infestans*, is associated with significant repellent responses, suggesting a defensive function (Audino et al. 2007). Sabinene, a monoterpene found in essential oils, has been shown to attract *Apolygus lucorum* in host plants such as *Artemisia lavandulaefolia* (Tian et al. 2023). β-Terpinene, another common monoterpene hydrocarbon found in many essential oils of plants, plays multiple ecological roles. For instance, in *Myrmicaria eumenoides*, β-terpinene, in combination with (+)-limonene, α-phellandrene and α-terpinolene, is part of a venom glands secreted blend that promotes rapid nestmate recruitment and coordinated colony responses (Yang et al. 2025).

Geranylacetone, detected exclusively in *B. davidii*, is an important monoterpene involved in insect attractant and plays a role in aggregation-sex pheromone systems. It serves as a key component in attracting the parasitoid *Mastrus ridibundus* (Müller and Buchbauer 2011) and as an aggregation-sex pheromone in *Arhopalus ferus* (Clavijo et al. 2024). It has also been used in traps for invasive yellowjackets (Babcock 2018). Finally, linalyl acetate, found only in *L. maculatum*, has been linked to pest attraction, for example in shade trees within tea plantations (Ghosh et al. 2021).

Some of the plants analysed in this study may exhibit fluctuations in their volatile profile depending on specific ecological factors or even throughout the diurnal cycle. However, the research has provided a comprehensive list of volatile compounds and highlighted their multifaceted role in mediating insect behaviour. These findings underline the potential of plant volatile as targets for eco-friendly pest management strategies, particularly in relation to *V. velutina*. With regard to terpenes, the results emphasise their chemical potential for the design and implementation of effective and efficient pest management strategies and possible environmental disasters.

The next phase of the study will focus on the attractant potential of the most frequently occurring volatile compounds through bait-trapping control method. To achieve this objective, further studies are required to assess the behavioural response of *V. velutina* to these volatile compounds. This should involve the use of an olfactometer or other appropriate methodology to quantify insect responses. Such studies will facilitate the determination of effective concentrations and compound combinations, which can then be tested in field experiments through their incorporation into bait-trapping systems.

## Conclusions

The plants visited by *V. velutina* were identified as foraging sites used for the acquisition of food sources (particularly, protein or carbohydrates). The flora observed comprised typical species found in the region of Galicia, although some cultivated species, notably those from the genus *Brassica*, also featured prominently. A total of 110 volatile compounds were identified as naturally occurring in the plant samples, spanning 20 chemical families. Amond these, terpene compounds were the most abundant, with linalool being the most predominant. A review of the literature identified some of these compounds as analogues of insect pheromones, such as nonanal and farnesene. These compounds may play a significant role in the communication of *V. velutina* and potentially act as attractants in a bait-trapping control method. Despite this, the extensive list of volatile compounds identified in this study also suggest the presence of numerous compounds capable of attracting a wide array of insect species. Consequently, the next phase of this research will involve the experimental evaluation of selected compounds for their potential application as trap baits in the integrated management of *V. velutina*.

## Data Availability

The data generated and/or analysed in the current study are available from the corresponding author upon reasonable request.
